# Nonoxidative coupling of methane to ethane over a Pd–Bi deposited titania photocatalyst in a flow reactor

**DOI:** 10.1039/d5sc09539e

**Published:** 2026-01-27

**Authors:** Preetam Dash, Yuan Zhong, Daichi Takami, Akira Yamamoto, Hisao Yoshida

**Affiliations:** a Graduate School of Human and Environmental Studies, Kyoto University Yoshida Nihonmatsu-cho, Sakyo-ku Kyoto 606-8501 Japan yoshida.hisao.2a@kyoto-u.ac.jp; b Department of Applied Chemistry, Faculty of Science and Engineering, Kindai University 3-4-1 Kowakae Higashiosaka Osaka 577-8502 Japan

## Abstract

Photocatalytic nonoxidative coupling of methane (photocatalytic NOCM) is a unique strategy for directly converting methane into more valuable C2 hydrocarbons like ethane and ethene at lower temperatures compared to traditional thermo-catalytic methods. In the current study, we developed a Pd–Bi/TiO_2_ photocatalyst prepared by a simultaneous photodeposition (PD) method. Under the present conditions, a stable ethane formation rate of 5.8 µmol h^−1^ was achieved with 99% ethane selectivity, which is significantly higher than that of previously reported photocatalysts, such as Au/TiO_2_ or Pd–Bi/Ga_2_O_3_ under the same reaction conditions. Part of the deposited Bi under PD conditions partially alloyed with Pd to form Pd–Bi alloy species in the case of the bi-metallic co-catalyst, which are proposed to be responsible for the higher selectivity towards ethane. The synergistic effect of Pd and Bi was studied experimentally.

## Abstract 2

A Pd–Bi bi-metallic co-catalyst offers highly selective non-oxidative coupling of methane over a TiO_2_ photocatalyst.

## Graphical abstract

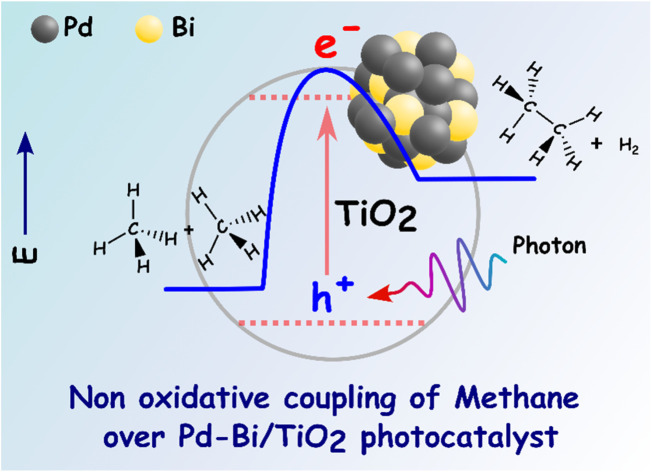

## Introduction

Methane is the primary component of natural gas. Due to its low cost and high energy content, it has long been utilized as a fuel. However, since it is not a renewable resource, its continued use appears undesirable considering current global environmental concerns. In contrast, methane is also the main constituent of biogas derived from biomass. This biomethane, being a renewable resource, aligns with the concept of carbon neutrality and should be effectively exploited. If considered as a future substitute for non-renewable resources like natural gas, coal, and petroleum, biomethane should be regarded not only as a fuel but also as a valuable carbon resource. However, it is a highly stable molecule with an average C–H bond energy of around 415 kJ mol^−1^.^1^ The complete oxidation of methane to CO_2_ is easy; however, partial oxidation to higher hydrocarbons is challenging due to thermodynamic limitations.

Among various methane conversion routes, catalytic non-oxidative coupling of methane (NOCM, eqn (1)) is a direct strategy to produce ethane and hydrogen without the use of oxygen and emission of carbon dioxide.^2,3^ Ethane is industrially more valuable than methane, and hydrogen is a well-known fuel. This makes NOCM a valuable approach for methane utilisation. Thermodynamically, however, the reaction is endothermic, resulting in a very low equilibrium conversion of 0.0002% at room temperature. Using a catalyst lowers the activation energy to increase the reaction rate while higher temperatures increase the equilibrium conversion to some extent.^4^ However, in thermo-catalytic NOCM, operating at temperatures above 1073 K often leads to catalyst deactivation^5^ because of methane decomposition (eqn (2)), a thermodynamically more favourable reaction that forms carbon deposits on the catalyst surface, making it inactive. Achieving NOCM at lower temperature is one of the possible solutions.

1 2CH_4_ → C_2_H_6_ + H_2_, Δ_r_*G*^*Θ*^_298K_ = 68.6 kJ mol^−1^

2 CH_4_ → C + 2H_2_, Δ_r_*G*^*Θ*^_298K_ = 50.7 kJ mol^−1^

Photocatalysis is an emerging green technology in catalysis since it proceeds even at lower temperatures such as room temperature. Using photon energy, NOCM can be achieved under ambient conditions as first reported by our group in 1998.^6^ The initial studies introduced “quantum photocatalysts”,^7,8^ which were insulators containing highly dispersed metal centres like Ti^4+^ and Ce^3+^ in a silica or silica-alumina matrix, serving as the active centres for photocatalytic NOCM.^9–11^ Later on, a new generation of modern photocatalysts^12–27^ for this reaction has gained attention. Among them, it is now well established that metal oxides like Ga_2_O_3_ (ref. 28), TiO_2_ (ref. 17 and 18) *etc.*, which are known as semiconductor photocatalysts, possess surface sites capable of activating methane upon light absorption. When their surface is modified with nanoparticles (NPs) of noble metals like Pd,^17^ Pt^19^ or Au,^18^ as a cocatalyst, their NOCM performance is enhanced by multiple factors. These metal-loaded photocatalysts are of particular interest because of their significantly higher activity and stability compared to the bare photocatalyst.

These noble metal NPs as cocatalysts are known to receive photoexcited electrons from semiconductor photocatalysts and thus reduce the recombination of photoexcited electrons and holes, significantly increasing the photocatalytic activity. However, their properties depend on various parameters such as their size, dispersion, and interaction with the semiconductor surface, which are deeply related to the deposition method and loading amount of the cocatalyst. For example, Lang *et al.* prepared various metal loaded (Ru, Rh, Pd, Au, Ag, Ir and Pt) TiO_2_ (P-25 Degussa) by a PD method and investigated their photocatalytic activity under simulated sunlight and found Au NPs to be the best cocatalyst for photocatalytic NOCM.^18^ They concluded that it was because of the efficient transfer of photogenerated electrons from TiO_2_ to Au NPs as evidenced by the potential drop across the M–TiO_2_ interface, which slowed down the carrier recombination. This resulted in a high ethane production rate of 81.7 µmol h^−1^ g_cat_^−1^ (0.41 µmol h^−1^). Recently, Longo *et al.* introduced Pd loaded TiO_2_ (P-25 Degussa) on a novel flow-through reactor reaching an ethane production rate of 1000 µmol h^−1^ g_cat_^−1^ (3.1 µmol h^−1^).^17^ In their observation, the preparation method highly affected the activity, with the strong electrostatic adsorption (SEA) method being better than the PD method. Although the experimental conditions differ considerably for both cases, making it difficult to determine which is superior, it is evident that the preparation method of the photocatalyst has a substantial influence on its performance.

Our recent research on photocatalytic NOCM employed a Pd–Bi bimetallic cocatalyst on a Ga_2_O_3_ photocatalyst prepared by a co-impregnation method, which exhibited a high ethane production rate reaching 1.1 µmol h^−1^ with a good selectivity of 97% in a flow reactor for a long reaction time such as 100 h.^29^ Therefore, it was concluded that Pd^0^, which was generated in the reductive conditions of photocatalytic NOCM, accepted the photogenerated electrons from the Ga_2_O_3_ photocatalyst, and BiO_*x*_ species prevented methane decomposition, thus improving catalyst stability in a long-term test. It was concluded that the two elements had independent contributions towards NOCM. In that work, although the Pd–Bi bimetallic cocatalyst prepared by an impregnation method was applied to a TiO_2_ photocatalyst, it resulted in poor performance and further examination has not been carried out in detail.^29^ In the present study, it was discovered that a Pd–Bi bimetallic cocatalyst, deposited on the TiO_2_ photocatalyst by a simultaneous PD method, achieved up to a fifty-fold increase in the C_2_H_6_ production rate compared to bare titania, reaching 5.8 µmol h^−1^ with excellent ethane selectivity, maintaining a stable production rate for at least 5.5 h.

## Experimental section

### Catalyst preparation

TiO_2_ (ST-01, 300 m^2^ g^−1^, anatase, Ishihara Sangyo Kaisha, Ltd) and TiO_2_ (AEROXIDE P-25, anatase and rutile, 55 m^2^ g^−1^, Evonik) were used without any further change. γ-Al_2_O_3_ powder (JRC-ALO-7, 180 m^2^ g^−1^) was supplied by the Catalysis Society of Japan. For metal loading with lower contents, a Pd standard solution (1 mg mL^–1^ PdCl_2_ in 1 M HCl aq.) and a Bi standard solution (1 mg mL^–1^ Bi(NO_3_)_3_ in 0.75 M HNO_3_ aq.) were used. For higher loading, PdCl_2_ (≥99%), Bi(NO_3_)_3_·5H_2_O (≥99.5%), methanol (≥99.5%), and NaOH aqueous solution (1 mol L^−1^) purchased from Nacalai Tesque Inc. were used.

The Pd–Bi cocatalysts were deposited on the TiO_2_ photocatalyst using a simultaneous PD method reported elsewhere.^30^ Since the redox potentials of Pd and Bi are 0.915 and 0.317 V *vs.* SHE, respectively, more positive than the bottom of the conduction band of TiO_2_ (*ca.* −0.5 V *vs.* SHE), they can be deposited on the TiO_2_ surface through reduction by photoexcited electrons (Fig. S1).^32–34^ Typically, 1.4 g of TiO_2_ powder (typically ST-01, unless otherwise specified) was dispersed in 250 mL of 10% methanolic aqueous solution in a borosilicate eggplant flask, followed by stirring for 15 min. Then, required amounts of the standard Pd and Bi aqueous solutions (1 mg mL^−1^) were added as metal precursors. The mixture was stirred in the dark for 30 min, followed by light irradiation from one side of the flask with a Xe lamp (300 W, Cermax-PE300BUV) for 1 h, where the light intensity measured at 365 nm wavelength using a UV radiometer (a Topcon UVR-300 equipped with a UD-360A detector) was 30 mW cm^−2^. The sample was filtered under suction, followed by washing with 500 mL of deionised water. It was then dried at 353 K overnight in an electric oven and collected. The sample was noted as *x*Pd–*y*Bi/TiO_2_, where *x* and *y* represent the weight% of Pd and Bi, respectively, with respect to titania. Single Pd and Bi loaded TiO_2_ samples (*x*Pd/TiO_2_ and *y*Bi/TiO_2_) were also prepared by the same method. For Pd and Pd–Bi samples with higher loading amounts, such as 5 and 7 wt%, the flask was sealed and purged with Ar before irradiation to ensure an inert environment, and all other procedures were carried out as described above except PdCl_2_ (5.29 mg mL^−1^ in 1 M HCl) and Bi(NO_3_)_3_ (10 mg mL^−1^ in 0.8 M HNO_3_) aqueous solutions prepared from the respective metal salts were used instead of the standard Pd and Bi (1 mg mL^−1^) solutions. It is worth mentioning that 0.1Pd/TiO_2_ and 0.1Pd–0.1Bi/TiO_2_ were also prepared by the PD method under an Ar atmosphere. However, as no noticeable differences in photocatalytic performance were observed compared to the corresponding samples prepared under air, these samples are not discussed separately.

The Pd–Bi loaded alumina was prepared by a deposition–precipitation and H_2_ reduction (DP-H_2_) method. Typically, 2 g of Al_2_O_3_ powder was dispersed in 100 mL of deionised water by ultrasonication for 15 min, followed by stirring for 30 min. Then, the required amounts of PdCl_2_ (5.29 mg mL^−1^ in 1 M HCl) and Bi(NO_3_)_3_ (10 mg mL^−1^ in 0.8 M HNO_3_) aqueous solutions were added as metal precursors and stirred for 30 min. The pH was increased to 11 by adding 1 M NaOH solution dropwise to the suspension. Then, the suspension was stirred for 24 h at 298 K. It was then filtered and washed 2 times with deionised water and then dried at 353 K overnight in an oven. The obtained powder was placed in a 20 mL test tube, which was sealed with a septum, and then Ar was purged to remove the air. It was then heated to 433 K and kept at this temperature for 30 min in a flow of Ar. Then, H_2_ was purged to replace Ar under heating conditions, which was continued for 45 min under a continuous H_2_ flow at 433 K to reduce the deposited Pd and Bi species.

### Photocatalytic NOCM experiment

The photocatalytic NOCM experiment was conducted in a flow reactor (Fig. S2). Typically, 1 g of catalyst was pressed under 40 MPa to form a pellet, which was then crushed into granules of 710–310 µm using a mesh filter (25–50 mesh). The granules were filled into a thin square quartz cell with dimensions of 2 × 2 × 0.1 cm^3^, the irradiation area was 4 cm^2^, and the cell is connected to two glass tubes on opposite sides. The weight of the granules filled in the cell was 0.52 g and 0.46 g for titania and alumina samples, respectively. In the previous study, it is elucidated that only one side of the granule surface can contribute to the photocatalytic reaction in this setup,^29^ which is the reason why we express the production rate without normalization by the catalyst weight by using the unit µmol h^−1^. The sample cell was purged with Ar at a flow rate of 27 mL min^−1^ for 30 min at 298 K to remove air from the cell. Subsequently, as a pretreatment, H_2_ was introduced into the feed gas at a flow rate of 3 mL min^−1^ (10 v/v% of the total feed gas) under irradiation for 30 min with the full spectrum of a Xe lamp (300 W, Cermax-PE300BUV), the intensity of which was measured using a UV radiometer mentioned above to be 10 mW cm^−2^ at 365 nm wavelength. The temperature of the cell increased to around 353 K upon irradiation, recorded using an infrared thermometer (TMHX-CGE2400) on the outside of the cell. This mild reductive pre-treatment was intended to clean the exposed catalyst surface from adsorbed organic impurities and to reduce the oxidised Pd and Bi species formed during the drying step in air. Even if the *ex situ* characterization mentioned later suggests the presence of an oxidised form of the cocatalyst, the working state in the photocatalytic reaction is expected to be metallic for both elements, Pd and Bi. After the pretreatment, the H_2_ flow was stopped, and methane was introduced at 3 mL min^−1^ (10% CH_4_ in Ar). The total feed gas had a contact time of around 0.8 s and a space velocity of 4500 h^−1^. After confirming that only methane and Ar were present in the feed, the cell was irradiated again with the same irradiation source to start the photocatalytic reaction. For the analysis of the products in the resulting gas mixture, two gas chromatograph equipped with different detectors, GC-TCD (Shimadzu, GC-8A, Ar carrier, molecular sieves: 5A, and column temperature: 333 K) and GC-FID (Shimadzu, GC-8A, Ar carrier, Gaskuropack 54, and column temperature: 363 K), were used. The GC-TCD could detect H_2_, O_2_, N_2_, CO, and CH_4_. Although CO_2_ may have been produced upon the reaction of methane with adsorbed water or the surface hydroxyls of TiO_2_ at the initial period of the experiment, it was not detected within the minimum detection range of 5 µmol h^−1^. The GC-FID could detect CH_4_, C_2_H_6_, C_2_H_4_, C_3_H_6_, and C_3_H_8_ under the provided conditions. In the present study, the observed products were almost limited to ethane and hydrogen, although a tiny amount of propane and ethylene was observed in a few cases. This is because the consecutive reactions are unlikely to proceed due to the small amount of product. Thus, the definition of the product ratio, *R*(C_2_H_6_/H_2_), showing the NOCM selectivity, is as follows (eqn (3)):3
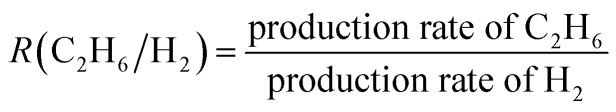


### Catalyst characterisation

The crystal phase of the samples was determined by powder X-ray diffraction (XRD) performed using an X-ray diffractometer (Lab X XRD-600, Shimadzu) under Cu-Kα (40 kV, 30 mA) radiation. Diffuse reflectance (DR) UV-vis spectra were obtained using a spectrometer (V-770, JASCO) with a BaSO_4_ plate as reference. Pd K-edge X-ray absorption spectra (XAS) were recorded using synchrotron radiation at NW10A, PF-AR KEK, Tsukuba, Japan, with a double Si(311) monochromator, where an ion chamber and a 21-element Ge-SSD detector were used in transmission and fluorescence modes, respectively. The Bi L_3_-edge XAS spectra were recorded at BL9A, PF KEK, with a double Si(111) monochromator, where an ion chamber and a 7-element Ge-SDD detector were used to record I intensity in transmission and fluorescence modes, respectively. The actual loading amounts of Pd and Bi in the photocatalyst samples were confirmed by X-ray fluorescence (XRF) using an EDX-8000 (Shimadzu). The TEM and STEM images and EDS mapping were taken with a JEM-ARM200F field emission microscope at 200 kV.

## Results and discussion

### Characterisation

**Fig. 1:**
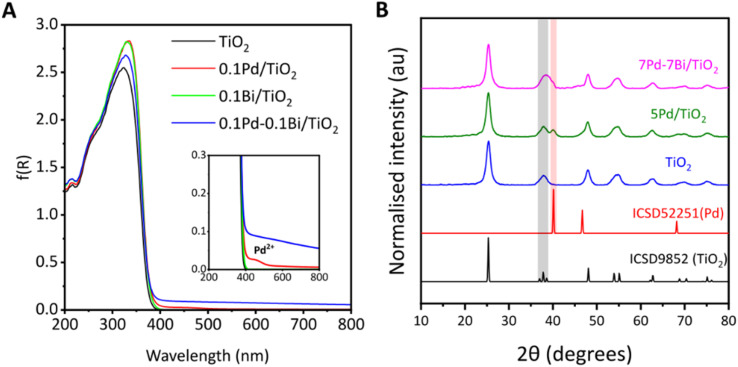
(A) DR UV-vis spectra converted to the Kubelka–Munk plot and (B) XRD profiles of the samples and references, where TiO_2_ (ST-01) was used. The reference XRD data were obtained from the Inorganic Crystal Structure Database (ICSD).

The DR UV-vis spectrum of the bare TiO_2_ sample showed a wide absorption band with an edge at 365 nm (Fig. 1A), indicating a band gap of 3.2 eV. After loading cocatalysts, the absorption edge of TiO_2_ remained unchanged, indicating no significant alteration of the band structure. The spectra of the TiO_2_ and 0.1Bi/TiO_2_ samples were almost identical. For 0.1Pd/TiO_2_, a very small shoulder peak observed around 460 nm was assignable to the d–d transition of the Pd^2+^,^31^ suggesting the presence of oxidised Pd species in the air-exposed sample. For the Pd loaded samples, 0.1Pd/TiO_2_ and 0.1Pd–0.1Bi/TiO_2_, an elevated baseline was observed with almost no dependence on the wavelength, which was likely due to a change in the scattering coefficient (*k*) of TiO_2_ due to the presence of metallic species. For 0.1Pd–0.1Bi/TiO_2_, a larger baseline lift indicates a higher metallic nature of the cocatalyst, also confirmed by XAS analysis as mentioned later.

The samples show XRD patterns assignable to anatase TiO_2_ (Fig. 1B). The diffraction pattern of Pd, Bi, or Pd–Bi species could not be observed at a lower loading of 0.1 wt% (not shown) probably due to the low content and high dispersion. However, when Pd and Pd–Bi were loaded at higher contents using the PD method, their diffraction lines became observable. For 5Pd/TiO_2_, a distinct diffraction peak at 40.1° corresponds to the (111) plane of Pd (ICSD 92251). For 7Pd–7Bi/TiO_2_, a broad diffraction peak at 37–40° was observed. There are no diffraction signals from α-Bi_2_O_3_ or PdO in the range of 37–40° (Fig. S3A), β-Bi_2_O_3_ has some overlap in that region but it is minor; therefore, the contribution should be small if it exists. Although a clear diffraction from the intermetallic phase could not be determined, it is possible that the diffraction lines from one or some intermetallic phases, such as Pd_3_Bi, Pd_8_Bi_3_, and Pd_5_Bi_2_, overlap in this broadened region (Fig. S3B). Therefore, the broadening likely resulted from the overlapping of the diffraction lines from the (103), (004), (112) planes of titania, (111) of Pd (shoulder), and one or some of the other intermetallic phases of Pd and Bi (Fig. S3B). Recently, Surya *et al.* prepared a Pd_3_Bi intermetallic compound on the surface of Ga_2_O_3_ by a PD method.^30^ As mentioned above, since the energy of the conduction band of anatase titania is high enough to reduce both Bi^32^ and Pd,^33,34^ the alloying of Pd–Bi is possible under PD conditions (Fig. S1). No Bi_2_O_3_ diffraction line was observed even though Bi L_3_-edge XANES suggested the existence of oxide like species in the air exposed sample (discussed later), suggesting the formation of a Bi_2_O_3_ amorphous phase when the samples are dried in the presence of air. Therefore, it is suggested that the Pd–Bi cocatalyst consisted of metallic, oxidised and alloy species in the current air-exposed sample.

**Fig. 2:**
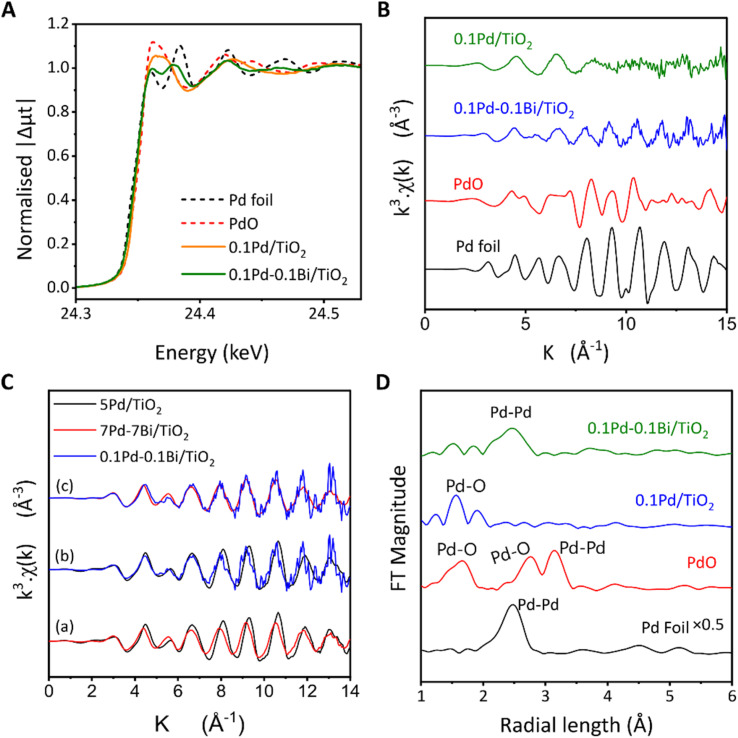
(A) The Pd K-edge XANES spectra of the Pd and Pd–Bi loaded TiO_2_ samples with references, (B) EXAFS spectra of the Pd and Pd–Bi loaded TiO_2_ samples, (C) a comparison of EXAFS spectra of the Pd–Bi loaded TiO_2_ sample with higher loading samples as references, and (D) the Fourier-transformed EXAFS spectra of the Pd and Pd–Bi loaded TiO_2_ samples with references.

The Pd K-edge XANES spectra of Pd and Pd–Bi loaded titania samples before the reaction test indicate that, for 0.1Pd/TiO_2_, Pd is predominantly in a similar local coordination state to PdO, suggesting that Pd was oxidised, whereas the spectrum of 0.1Pd–0.1Bi/TiO_2_ is rather close to that of metallic Pd although not identical (Fig. 2A). The linear component fitting of the XANES spectra of 0.1Pd/TiO_2_ suggested that the spectra consisted of 26% Pd^0^ and 74% Pd^2+^ (Fig. S4). For 0.1Pd–0.1Bi/TiO_2_, the Pd–K XANES spectra consisted of 73% Pd^0^ and 27% Pd^2+^ (Fig. S5A) and Bi-L_3_ XANES spectra consisted of 21% Bi^0^ and 79% Bi^3+^ (Fig. S5B). This indicated that the atomic ratio of Pd^0^ : Bi^0^ was 6.8 : 1 in 0.1Pd–0.1Bi/TiO_2_. Additionally, 0.1Pd/TiO_2_ and 0.1Pd–0.1Bi/TiO_2_ contained 26% and 73% of loaded Pd in a metallic state (Pd^0^), respectively, which indicates that the presence of Bi species preserves the metallic state of Pd species. The EXAFS spectra (Fig. 2B) show that the two samples have a smaller amplitude than the metal foil, suggesting a lower coordination number originating from small size or disorder due to low crystallinity of the metal nanoparticles. The spectra of 0.1Pd/TiO_2_ become increasingly noisy with increasing *k* value due to the lack of strongly scattering heavy atoms around Pd and a very low wt%. Since the local coordination of Pd changes in the nanoparticle form, and the coexistence of Pd and PdO further complicates the EXAFS spectra, comparison with Pd foil is not very reliable for changes due to alloying in the nanoparticle. A more appropriate reference could be 5Pd/TiO_2_ with high enough Pd loading, which can also be confirmed by XRD (Fig. 1B). When EXAFS spectra of 5Pd/TiO_2_ and 7Pd–7Bi/TiO_2_ were compared (Fig. 2C(a)), a clear phase shift of the EXFAS oscillation could be observed starting from 4 Å^−1^, which was due to changes in the bond lengths due to Bi incorporation into the Pd local structure; additionally, the slightly smaller amplitude suggests a higher disorder of the Pd species in the 7Pd–7Bi/TiO_2_ sample due to mixed phases of Pd and other intermetallic compounds with Bi. 0.1Pd–0.1Bi/TiO_2_ shows a more similar oscillatory feature to 7Pd–7Bi/TiO_2_ (Fig. 2C(b and c)), suggesting that both the bimetallic samples have similar local environments irrespective of the loading amount. Upon Fourier transformation (FT), the peak at *r* = 1.65 Å is assignable to Pd–O for 0.1Pd/TiO_2_, while the one at *r* = 2.5 Å for 0.1Pd–0.1Bi/TiO_2_ is Pd–M (M = Pd or Bi) (Fig. 2D). This clearly indicates that Pd exists in an oxide like environment in 0.1Pd/TiO_2_, whereas it predominantly forms metallic nanoparticles in 0.1Pd–0.1Bi/TiO_2_. For the former, 0.1Pd/TiO_2_, it is possible that a large fraction of nanoparticles of Pd were oxidised during the drying step. However, many previous studies suggest that the Pd species is reduced under photocatalytic NOCM conditions to be active sites for NOCM.^29,35^ In our case, the decrease of the d–d transition band of Pd^2+^ and the increase of the baseline due to the formation of metallic Pd species in the Kubelka–Munk spectra (Fig. S6) for the spent 0.1Pd/TiO_2_ further support the reduction of the cocatalyst under the working conditions.

The analysis of Bi L_3_-edge XANES indicated the presence of oxide-like (Bi_2_O_3_) structures in the air-exposed samples (Fig. S7). In the case of 7Pd–7Bi/TiO_2_, the absorption edge was slightly at lower energy than that for Bi_2_O_3_, suggesting slightly less charge on Bi. The Bi-L_3_ EXAFS Fourier transform analysis of 0.1Pd–0.1Bi/TiO_2_ was difficult due to low oscillation intensity compared to the noise level (low S/N ratio) but for 7Pd–7Bi/TiO_2_ in addition to the scattering at *r* = 1.6 Å corresponding to the Bi–O bond, another scattering at *r* = 2.5 Å was observed, which will be discussed later (Fig. S8).

**Fig. 3:**
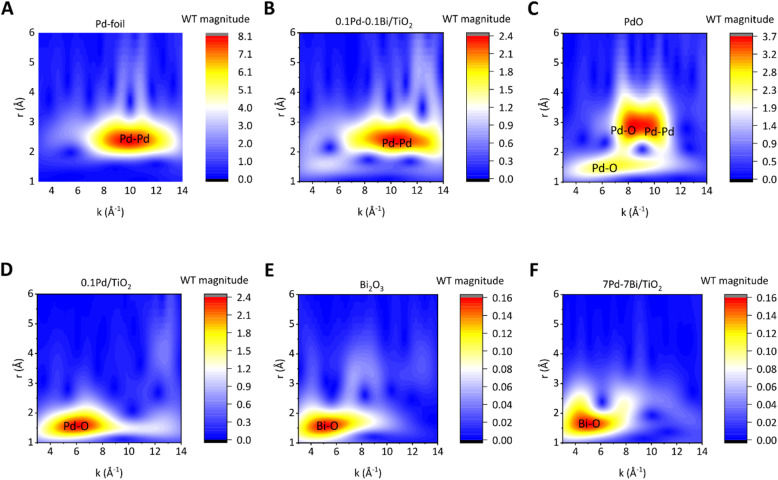
The wavelet transform analysis of Pd K-edge EXAFS for the samples: (A) Pd foil, (B) 0.1Pd–0.1Bi/TiO_2_, (C) PdO, and (D) 0.1Pd/TiO_2_, and those of Bi L_3_-edge XAFS for the samples (E) Bi_2_O_3_ and (F) 7Pd–7Bi/TiO_2_.

The EXAFS wavelet transform indicates that the air-exposed 0.1Pd–0.1Bi/TiO_2_ sample possesses a metallic environment similar to that of Pd foil (Fig. 3A and B). However, its WT magnitude is lower (see the scale on the right), which can be attributed to the smaller amplitude of the EXAFS spectra discussed previously in Fig. 2B. The wavelet transform map is more spread along the *x* axis (wave vector, *k*) due to the interaction of Pd with lighter O and heavier Bi atoms. The WT spectrum of air-exposed 0.1Pd/TiO_2_ matches with that of PdO except that there is no scattering from the second shell of O or Pd due to the highly dispersed state and small loading on TiO_2_ (Fig. 3C and D). From the Bi L_3_-edge EXAFS-WT analysis (Fig. 3E and F) of air-exposed 7Pd–7Bi/TiO_2_, it can be realised that the FT scattering at 2.5 Å consists of two wave-vector (*k*) components at 4–5 Å^−1^ and 8 Å^−1^ for air-exposed 7Pd–7Bi/TiO_2_ (Fig. 3F). According to EXAFS theory, heavier neighbouring atoms exhibit stronger oscillations at larger *k* values, whereas lighter atoms show stronger oscillations at smaller *k* values in EXAFS spectrum. Therefore, heavier atoms have strong influence on the FT spectra at a higher value of the wave vector (*k*) which is visualised in WT. Upon careful inspection and comparison with the WT of Bi_2_O_3_, it is expected that the *k* component at 8 Å^−1^ can be attributed to a heavy atom like Pd or Bi. Since the length of 2.5 Å is too short to be attributed to the second shell of Bi_2_O_3_, it must be associated with a metal in the first shell. This partly supports the alloying of Bi with Pd.

The XAFS curve fitting analysis of 5Pd/TiO_2_ and 7Pd–7Bi/TiO_2_ was conducted and a set of parameters were obtained, which are summarised in Table S2. For 7Pd–7Bi/TiO_2_, the two first-shell models (Pd–Pd and Pd–Bi) yield a good fit with a low *R*-factor; however, the coordination number associated with Pd–Bi had a large uncertainty of 4.4 ± 16 and showed strong correlation with a DW factor of ≈0.95. This suggests that the Pd–Pd and Pd–Bi bond lengths do not differ sufficiently to be uniquely separated by the curve fitting analysis.

**Fig. 4:**
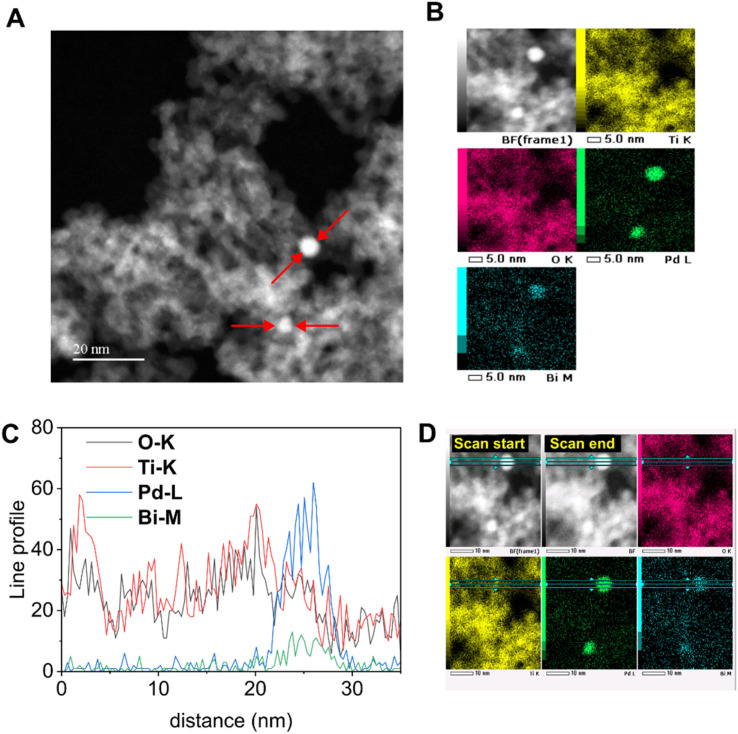
(A) STEM image and (B) STEM-EDS elemental maps, and (C and D) EDS-line profile analysis of the 0.1Pd–0.1Bi/TiO_2_ sample.

The TEM image of air-exposed 0.1Pd–0.1Bi/TiO_2_ shows aggregated titania nanoparticles of 7 nm size and some dark contrast spots that can possibly be attributed to Pd–Bi nanoparticles (Fig. S9A). Although clear lattice fringes of metallic nanoparticles were not resolved, fast Fourier transform analysis of these dark spots reveals a dominant diffraction spacing of *d* = 0.112 nm, which may correspond to high-index planes of a metallic phase (Fig. S9B). The STEM images of 0.1Pd–0.1Bi/TiO_2_ show metallic nanoparticles of around 3–5 nm diameter loaded over titania (Fig. 4A). EDX elemental mapping indicates that Pd and Bi are homogeneously distributed within the nanoparticles (Fig. 4B). Although a higher reduction potential of Pd^2+^ relative to Bi^3+^ suggests that Pd would reduce first, followed by Bi deposition that could form a bismuth shell, subsequent air exposure would be expected to yield a Pd core-BiO_*x*_ structure. However, line profile analysis of the nanoparticle suggested that the Pd–Bi species are metallic due to the absence of an oxygen signal from those particles (Fig. 4C and D). Therefore, the formation of a distinct Pd-core/BiO_*x*_ shell structure is unlikely. However, the presence of a compositional gradient within the nanoparticles, such as a Pd-enriched core with a Pd–Bi-enriched surface region, cannot be excluded. Since the presence of Bi oxide species is evidenced in the air-exposed sample by XAFS analysis, the observed Bi atoms in the metallic nanoparticles would only be a fraction of the entire Bi species loaded on TiO_2_.

### Photocatalytic activity test

**Fig. 5:**
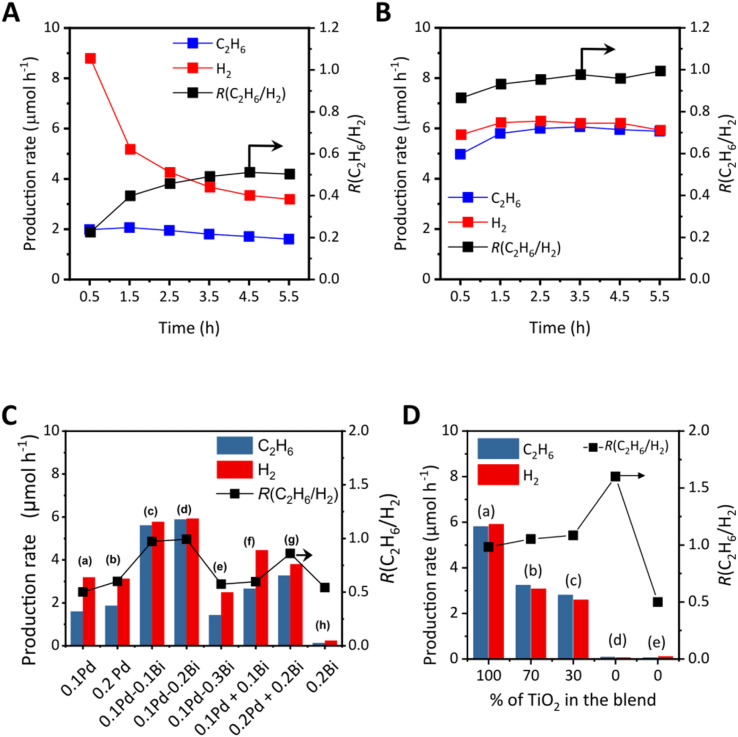
(A) Time courses of ethane (blue) and hydrogen (red) production rates over 0.1Pd/TiO_2_, (B) time courses of ethane (blue) and hydrogen (red) production rates over 0.1Pd–0.1Bi/TiO_2_, (C) production rates at 5.5 h over various samples, and (D) those over (a) 0.1Pd–0.1Bi/TiO_2_, (b and c) two blended catalysts consisting of TiO_2_ and Pd–Bi/Al_2_O_3_, and (d and e) two Pd–Bi/Al_2_O_3_ samples (see Table 1 for the details of the samples in (D)). The black line with square symbols corresponds to the second y axis, which represents *R*(C_2_H_6_/H_2_). The ideal value of *R*(C_2_H_6_/H_2_) is 1, which corresponds to 100 % NOCM selectivity.

Fig. 5A shows the time course of the production rate of ethane and hydrogen with the ratio of them, *R*(C_2_H_6_/H_2_), over the 0.1Pd/TiO_2_ photocatalyst in the flow reactor. Compared to bare titania that could not promote photocatalytic NOCM (Fig. S10A) and be reduced to show a blue colour due to reduction (Fig. S10B and C), the Pd-modified titania had a much higher production rate of ethane and hydrogen (Fig. 5A). At 5.5 h, 0.1Pd/TiO_2_ produced ethane and hydrogen at rates of 1.6 µmol h^−1^ and 3.2 µmol h^−1^, respectively, where H_2_ production exceeded C_2_H_6_, suggesting that this TiO_2_ photocatalyst with a Pd cocatalyst promoted methane coupling (eqn (1)) and other side reactions, possibly methane decomposition (eqn (2)). The decline in hydrogen production over time likely resulted from the deactivation of methane decomposition sites. On the other hand, with 0.1Pd–0.1Bi/TiO_2_, ethane and hydrogen production remained stable for at least 5.5 h, reaching much higher production rates for ethane and hydrogen, 5.8 µmol h^−1^ and 5.9 µmol h^−1^, respectively (Fig. 5B).

Fig. 5C shows the production rates and *R*(C_2_H_6_/H_2_) with various photocatalysts after 5.5 hours from the start of the reaction test in the flow reactor. Increasing the Pd loading from 0.1 wt% to 0.2 wt% in the *x*Pd/TiO_2_ samples did not significantly affect the activity (Fig. 5C(a and b)), suggesting that a small change in Pd loading has a negligible effect on ethane formation. The 0.1Pd–*y*Bi/TiO_2_ samples including moderate amounts of Pd and Bi (*y* = 0.1 or 0.2 wt%) exhibited much higher activity and selectivity for NOCM than the *x*Pd/TiO_2_ samples, while the activity and selectivity decreased when the Bi loading was 0.3% (Fig. 5C(c–e)). This indicates that the coexistence of Pd and Bi cocatalysts in adequate amounts enables highly selective performance for NOCM. The physical mixture of the Pd/TiO_2_ and Bi/TiO_2_ samples had reduced activity (Fig. 5C(f and g)) compared with the active 0.1Pd–*y*Bi/TiO_2_ samples (*y* = 0.1 or 0.2), showing that the presence of Pd and Bi species on the same TiO_2_ particles would be preferable. In addition, it is noted that the mixture showed still obviously higher activity than the single Pd loaded catalysts (Fig. 5C(a and b)). Therefore, although the alloy formation in the active 0.1Pd–*y*Bi/TiO_2_ samples would improve the activity and selectivity, Pd and Bi can still independently function as cocatalysts to improve the selectivity even if they are present at different sites on the titania shown here, as suggested in the previous work on the Ga_2_O_3_ photocatalyst.^29^ In contrast, 0.2Bi/TiO_2_ showed very low activity (Fig. 5C(h)), indicating the presence of the Pd cocatalyst is required for the high performance in photocatalytic NOCM.

### Cocatalyst–photocatalyst interaction

To properly understand the catalytic behaviour of the Pd–Bi nanoparticles we tested a blended catalytic system, which is a physical mixture of the TiO_2_ photocatalyst and a supported metal catalyst, *i.e.*, Pd–Bi loaded Al_2_O_3_. Two such blended catalysts were implemented where the contents of Pd and Bi in the physical mixture were at similar levels, *ca.* 0.1 wt% and 0.1 wt%, respectively, to the 0.1Pd–0.1Bi/TiO_2_ photocatalyst with different TiO_2_ contents except for the Pd–Bi/Al_2_O_3_ sample (see Table 1). The deposition–precipitation approach was used to load the co-catalysts onto Al_2_O_3_ since PD cannot be used for alumina, which is not a photocatalyst. The obtained catalyst contained metal nanoparticles of *ca.* 5 nm diameter. (Fig. S11). The STEM-EDS analysis of a nanoparticle also suggested the formation of Pd–Bi bimetallic nanoparticles (Fig. S12). Fig. 5D shows a comparative study of the two blended catalysts with the Pd–Bi/TiO_2_ photocatalyst and the Pd–Bi/Al_2_O_3_ catalyst. When compared to Pd–Bi/TiO_2_ (Fig. 5D(a)), the blended catalysts (Fig. 5D(b and c)) have less activity. However, both blended catalysts containing almost the same loading of cocatalysts with different TiO_2_ contents have almost comparable activity, even though the amount of photocatalyst (TiO_2_) is different in both cases (0.35 g for the former and 0.15 g for the latter, respectively). Both the 0.5Pd–0.33Bi/Al_2_O_3_ and 0.2Pd–0.14Bi/Al_2_O_3_ catalysts without the TiO_2_ photocatalyst had negligible activity (Fig. 5D(d and e)). Based on these observations, three mechanistic implications can be inferred:

**Table 1:** Contents of co-catalysts in the samples used for the experiments shown in Fig. 5D; the total catalyst amount was 0.5 g in each run

Entry	Sample	Content of TiO_2_ (g)	Contents of Pd and Bi, *x*Pd–*y*Bi (wt%)	
			To Al_2_O_3_	To the sample in the cell
1	Pd–Bi/TiO_2_	0.5	—	0.1Pd–0.1Bi
2	70% TiO_2_ + 30%Pd–Bi/Al_2_O_3_	0.35	0.5Pd–0.33Bi	0.15Pd–0.1Bi
3	30% TiO_2_ + 70%Pd–Bi/Al_2_O_3_	0.15	0.2Pd–0.14Bi	0.14Pd–0.1Bi
4	Pd–Bi/Al_2_O_3_	0	0.5Pd–0.33Bi	0.5Pd–0.33Bi
5	Pd–Bi/Al_2_O_3_	0	0.2Pd–0.14Bi	0.2Pd–0.14Bi

(i) When compared to the first three samples, the decline in the activity implies the significance of Pd–Bi species as a co-catalyst deposited on the TiO_2_ surface to improve the charge separation. When supported on Al_2_O_3_, the Pd and Bi species have limited contact with TiO_2_, limiting the activity. Since both TiO_2_ and Pd–Bi loaded Al_2_O_3_ were mixed and pressed together for granulation, there must be the possibility of contact of Pd–Bi with TiO_2_; however, the chance is much more limited compared to them directly loaded on TiO_2_.

(ii) Secondly, the comparison between the two blended samples signifies the effect of Pd–Bi as a metal catalyst, even though they are supported on a photocatalytically inactive material, Al_2_O_3_. The active radical intermediates generated on the surface of the TiO_2_ photocatalyst (*i.e.*, the methyl and hydrogen radicals, eqn (4) and (5)) have to migrate to the metal nanoparticles on the Al_2_O_3_, where they couple to form the products (eqn (6) and (7)). These radical species have been reported to migrate relatively long distances, on the order of millimetres in the gas phase and can also survive in the liquid phase in our previous works.^38–41^ Hence, the Pd–Bi species on Al_2_O_3_ function as a catalyst to promote the radical–radical coupling reaction, which is not a photocatalytic process but a dark process. The identical activity of the blended systems (Fig. 5D(b and c)) indicates that the amount of TiO_2_ in either case is saturated, meaning that there are enough photocatalytic sites for methane activation in both cases; therefore, there is no linear dependence of the weight of TiO_2_ and the photocatalytic activity under the current conditions. The Pd–Bi catalyst determines the production rate in photocatalytic NOCM. In other words, the rate determining process (RDS) under these conditions is the catalysis on the Pd–Bi species for radical–radical coupling.

For the further study of this radical migration pathway, the blended catalyst was introduced into the reaction cell *via* two different ways. First, TiO_2_ and Pd–Bi/Al_2_O_3_ were mixed and then granulated (Fig. S13b); second, TiO_2_ and Pd–Bi/Al_2_O_3_ were granulated separately (Fig. S13c). The size of each granule is 0.3–0.7 mm (25–50 mesh) in diameter, which is quite large for electron transfer between those particles even if they are in contact. When granulated separately, the activity obviously decreased significantly compared to the situation where TiO_2_ and Pd–Bi/Al_2_O_3_ are present in the same granules. However, when compared to bare TiO_2_ or Pd–Bi/Al_2_O_3_ (Fig. S13a, c and d), the ethane production was significantly higher (*ca.* 8 times higher than that of bare TiO_2_). In such a case, the methyl radicals generated on TiO_2_ particles should have migrated to the Pd–Bi sites on the contacted Al_2_O_3_ particles and coupled together. This result further strengthens the argument for migration of radical species from TiO_2_ to Pd–Bi/Al_2_O_3_. The *R*(C_2_H_6_/H_2_) values in (a) and (c) are much greater than 1 in Fig. S13; this suggests that without the presence or proper contact of the co-catalyst in the TiO_2_, H_2_ evolution is difficult in both cases due to its consumption for the reduction of TiO_2_.

(iii) It is also worth mentioning that both blended catalysts have higher activity and selectivity compared to single Pd loaded TiO_2_ (Fig. 5C(a and b)), leading to the third important conclusion that the synergy between Pd and Bi is not specific to supports like Al_2_O_3_, TiO_2_ or previously reported Ga_2_O_3_.^29^ This could be further verified when only Pd was employed as the metal catalyst instead of Pd–Bi for the blended catalytic system (Fig. S14).

The Pd and Bi species are recognized as bifunctional materials: one role is as cocatalysts for photocatalysts to enhance the charge separation of the photogenerated electrons and holes as discussed in (i) and the other role is as catalysts to promote homocoupling of radical species, methyl radicals and hydrogen radicals to form products, ethane and hydrogen as discussed in (ii). However, it should be noted that these metal species are also likely to promote non-productive cross-coupling reactions, *i.e.*, the reaction between methyl and hydrogen radicals, which do not yield NOCM products, ethane and hydrogen (eqn (1)), but instead lead to methane formation (eqn (8)).

Although the DP-H_2_ method was also applied to prepare 0.1Pd–0.1Bi/TiO_2_, the resulting catalyst performed poorly (Fig. S15). The *R*(C_2_H_6_/H_2_) value exceeded 1. Although the activity was higher, the trend was similar to that of Pd–Bi/TiO_2_ prepared by an impregnation method in our previous study where the C_2_H_6_ production rate was 0.4 µmol h^−1^ and H_2_ formation was not detected.^29^ Therefore, the PD method employed in the present study is a better approach compared to the impregnation and DP-H_2_ method for preparing Pd–Bi/TiO_2_ photocatalysts.

### The effect of light intensity

**Fig. 6:**
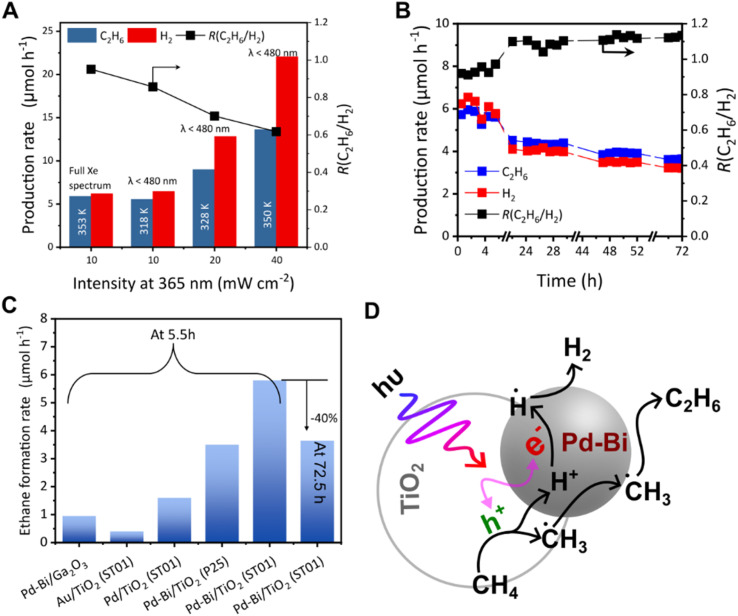
(A) The effect of light intensity on the photocatalytic NOCM over 0.1Pd–0.1Bi/TiO_2_ (after 2.5 h irradiation under flow), (B) a photocatalytic NOCM stability test for 72 h over 0.1Pd–0.1Bi/TiO_2_, (C) comparison of ethane production rates over different catalysts under the present reaction conditions, and (D) plausible mechanism of photocatalytic NOCM over the Pd–Bi/TiO_2_ photocatalyst.

The effect of light intensity on photocatalytic NOCM was investigated using a flow reactor (Fig. 6A). The light intensity was measured at 360 nm by using a UV radiometer. To limit the thermal effects induced by the photoirradiation, a cold mirror was employed. The mirror reflected lights of *λ* ≤ 480 nm changing the spectral distribution of the Xe lamp to mostly UV and blue light (Fig. S16A), which was used for the photocatalytic activity test (Fig. S16B). The two conditions of the same light intensity in the range of *λ* < 480 nm, but with or without light of *λ* > 480 nm, gave identical activity although the temperature was different (320 K and 350 K respectively in Fig. 6A), indicating that UV absorption of TiO_2_ drove the reaction. As expected, the production rate increased with increasing light intensity (Fig. 6A). However, the *R*(C_2_H_6_/H_2_) value decreased with an increase in the UV light intensity (Fig. 6A). With a gradual increase in the UV intensity, no other hydrocarbons such as C_2_H_4_ and C_2_H_2_ and steam reforming products such as CO could be detected within our detection range, which would otherwise result in a higher H_2_ production rate. This suggested that at higher light intensity, the methane decomposition (eqn (2)) takes place in addition to methane coupling (eqn (1)). The temperature was as high as that under low light intensity full spectrum conditions. Thus, methane decomposition as a side reaction is promoted by high light intensity rather than high temperature; in other words, lower light intensity is favourable for selective photocatalytic NOCM.

### Long term test

A long-term photocatalytic reaction test for 72 h was conducted over the best performing photocatalyst, 0.1Pd–0.1Bi/TiO_2_. Although the performance of the catalyst in the 5.5 h range was good with high selectivity, the photocatalytic activity unfortunately decreased to about 40% of the initial activity (Fig. 6B). The ratio *R*(C_2_H_6_/H_2_) slowly increased over 1 in 24 h and seemed to reach 1.2, indicating that the proton reduction to hydrogen slowed down in the long-term test. No significant carbon deposition could be observed from the Raman spectra after the 72 h test (Fig. S17). Both these results suggest that the primary reason for the deactivation is possibly not carbon deposition. Although a clear reason for this deactivation could not be obtained, the results suggest that the deactivation is possibly related to the change in the TiO_2_ surface upon long-term testing. Bare TiO_2_ under NOCM conditions turns blue suggesting the formation of the reduced titania (Fig S7B and C). This colour change is a common phenomenon observed in photocatalytic reactions in the presence of a hole scavenger like methanol.^36^ When the loading amount of the co-catalyst was increased to 0.5 wt%, the stability increased and the *R*(C_2_H_6_/H_2_) value was <1 and ≈1 for 0.5Pd/TiO_2_ and 0.5Pd–0.5Bi/TiO_2_, respectively (Fig. S18A and B). In this case, the gradual reduction in H_2_ production was not observed over the 72 h period. However, the activity was lower, possibly due to over-coverage of metal nanoparticles on TiO_2_. It is notable that even after 72 h, the activity of 0.1Pd–0.1Bi/TiO_2_ is significantly higher than the single Pd/TiO_2_ catalyst.

Other previously reported photocatalysts, Pd–Bi/Ga_2_O_3_ ^29^ and Au/TiO_2_,^18^ were also prepared and examined for the photocatalytic NOCM test under the same conditions, exhibiting less activity than the 0.1Pd–0.1Bi/TiO_2_ in terms of ethane production (Fig. 6C). For a comparison, the Pd–Bi cocatalyst (0.1 wt% each) was loaded on the benchmark Evonik P25 TiO_2_ by the PD method. Compared to ST01, it had a lower production rate of ethane at 5.5 h, which may be due to the lower surface area (81% lower than ST01) (Fig. 6C). Among these metal-loaded photocatalysts, the present 0.1Pd–0.1Bi/TiO_2_ photocatalyst exhibited the best performance in the current reaction conditions.

### Reaction mechanism

Based on the experimental observations, we tentatively propose a mechanism of NOCM over the Pd–Bi/TiO_2_ photocatalyst (Fig. 6D). Adsorbed methane is activated by the holes generated on TiO_2_ to form methyl radials (˙CH_3_) and protons (H^+^) (eqn (4)). The methyl radicals could migrate to metallic Pd–Bi NPs and be stabilised on the NP surface, where the presence of Bi species would contribute to maintaining the metallic state of the Pd–Bi NPs, as evidenced by XAFS (Fig. 2A), and possibly weaken the strong interactions between Pd NPs and the adsorbed methyl radicals, consequently promoting coupling of methyl radicals to form ethane (eqn (6)) and preventing methane decomposition (eqn (9)). It is reported that Bi contributes to high selectivity in the thermal NOCM reaction with the Pt catalyst supported on ZSM-5 zeolite.^37^ On the other hand, the photogenerated electrons are accepted by metallic Pd–Bi NPs, where they are utilised for proton reduction to hydrogen (Fig. 6D). Other Bi species might be present in the Pd–Bi/TiO_2_ photocatalyst as remaining Bi oxide species, the role of which will be discussed later.

### Role of cocatalysts

The performance of the physical mixture of TiO_2_ and Pd–Bi/Al_2_O_3_ supports the above reaction mechanism. The methyl radicals generated on the TiO_2_ photocatalyst can migrate to the Pd–Bi NPs on the photocatalytically inert support, Al_2_O_3_, where they couple to form ethane (Fig. 5C and S13). The hydrogen radicals generated on the TiO_2_ photocatalyst form H_2_ species either over the TiO_2_ surface or at the metal surface. However, the metal surface is important for the coupling of hydrogen radicals^41^ (as seen in Fig. S13a and c). Therefore, Pd or Pd–Bi is the catalytic site for radical–radical coupling of methyl or hydrogen radicals to form ethane or hydrogen, respectively.

The activity trends observed for the blended catalyst systems provide further insights that TiO_2_ serves as the primary site for methane activation and methyl radical generation, but efficient C–C coupling requires close spatial proximity to Pd–Bi. Specifically, the catalytic performance decreases in the order Pd–Bi/TiO_2_ (Fig. 5D(a)) > Pd–Bi/Al_2_O_3_ + TiO_2_ with intra-granular contact (Fig. S13b) > Pd–Bi/Al_2_O_3_ + TiO_2_ with inter-granular contact (Fig. S13c), indicating that increasing physical separation between TiO_2_ and Pd–Bi suppresses productive coupling. This trend suggests a limited diffusion length of the generated radical species. An increased separation leads to *R*(C_2_H_6_/H_2_) values exceeding unity (Fig. S13). This suggests that the generated proton as a result of methane activation might have partly been trapped over the TiO_2_ surface due to its positive charge. Together, these observations indicate that the effective methane activation site is located at, or very near, the Pd–Bi/TiO_2_ interface.

The physical mixture of Pd/TiO_2_ and Bi/TiO_2_ exhibited higher activity than single Pd/TiO_2_ or Bi/TiO_2_ samples in photocatalytic NOCM (Fig. 5C(f and g)). This result suggests an additional function of BiO_*x*_ species except for the stabilising metallic state of the Pd cocatalyst. Compared with bare TiO_2_ (Fig. S10A), 0.1Pd/TiO_2_ showed greatly increased activity, indicating that Pd promotes photocatalytic formation of methyl radicals (Fig. 5C(a and b)). In contrast, 0.2Bi/TiO_2_ exhibits a negligible contribution in this step (Fig. 5C(h)). Therefore, it relies on 0.1Pd/TiO_2_ for the methane activation step. Small variation in Pd loading 0.1 wt% or 0.2 wt% has little influence on the NOCM selectivity (Fig. 5C(a and b)). A comparison of the two 0.1Pd/TiO_2_ + 0.1Bi/TiO_2_ mixtures thus suggested that the amount of Bi changed the reaction selectivity (Fig. 5C(f and g)), *i.e.*, Bi species promote NOCM more selectively. When compared to the results with the same amount of Pd cocatalyst in the cell shown in Fig. 5C and Table 2 a and g, a larger loading of Bi species in the mixed sample increased the NOCM selectivity, *i.e.*, high *R*(C_2_H_6_/H_2_) (Fig. 5C(g)), which is supported by our previous work of NOCM with the Pd–Bi/Ga_2_O_3_ photocatalyst.^29^ This effect is attributed to the catalytic promotion of radical–radical coupling on Bi species (eqn (6)), which competes with and suppresses the successive photocatalytic oxidation of methyl radicals (eqn (9)). Since Bi/TiO_2_ could not exhibit NOCM activity if it worked alone, the photocatalytic activity of bare TiO_2_ to form methyl radicals would be suppressed by loading Bi species. The Bi/TiO_2_ moiety would be less active as a photocatalyst, but it works as a catalyst for the radical–radical coupling of methyl radicals (eqn (6)).

**Table 2:** Estimated amounts of co-catalyst in the cell used for the experiments shown in Fig. 5C, where the total catalyst amount was 0.52 g in each run

Entry	Sample name	Co-catalyst amount in the cell (mg)	
		Pd	Bi
a	0.1Pd/TiO_2_	0.52	—
b	0.2Pd/TiO_2_	1.04	—
c	0.1Pd–0.1Bi/TiO_2_	0.52	0.52
d	0.1Pd–0.2Bi/TiO_2_	0.52	1.04
e	0.1Pd–0.3Bi/TiO_2_	0.52	1.56
f	0.1Pd/TiO_2_ + 0.1Bi/TiO_2_	0.26	0.26
g	0.2Pd/TiO_2_ + 0.2Bi/TiO_2_	0.52	0.52
h	0.2Bi/TiO_2_	—	1.04

An additional conclusion is that the radical–radical coupling reactions preferably take place on the surface of metallic Pd, Bi, or Pd–Bi alloys. This is indirectly supported by our previous study, in which hydrogen radical coupling to form H_2_ molecules was catalysed by a metallic Pt NP catalyst.^41^ Further, it is notable that the cocatalyst of the photocatalyst can contribute to photocatalytic activity in multiple steps: first, as a receiver of the photoexcited electrons from the photocatalyst to avoid the electron–hole recombination and enhance the photocatalytic activity; second, as a promoter of the reduction reaction by the excited electrons; and third, through its catalytic properties that enhance radical–radical coupling between hydrogen radicals or methyl radicals to form hydrogen and ethane, which are the products of NOCM (eqn (1)).4
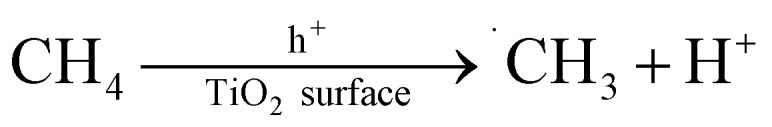
5
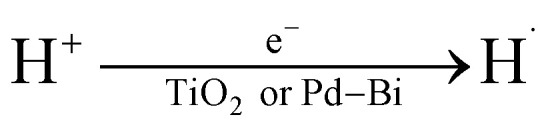
6
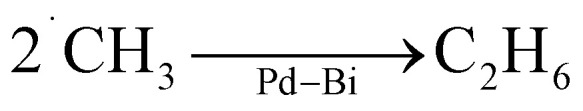
7
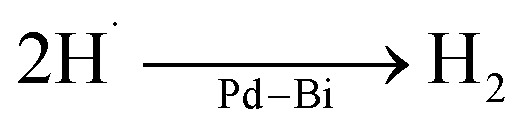
8
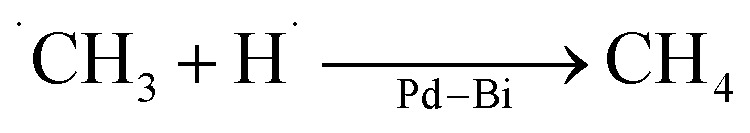
9
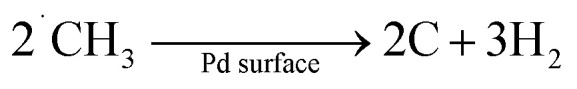


## Conclusions

Bimetallic Pd–Bi/TiO_2_ was successfully prepared by a simultaneous PD method. Simultaneous addition of Pd and Bi to the TiO_2_ photocatalyst improved the photocatalytic activity for ethane production, *ca.* 3 times higher than that of Pd/TiO_2_. Various characterisation studies suggested that Bi can partly alloy with Pd by the PD method, while some free Bi species exist as oxide-like species. Since high light intensity promotes side reactions, which result in lower *R*(C_2_H_6_/H_2_) translating to lower ethane selectivity, a low to moderate light intensity (<15 mW cm^−2^) is preferred for selective photocatalytic NOCM. The best Pd–Bi/TiO_2_ photocatalyst, however, suffered deactivation over a long-term test for 72 h possibly because of changes in the surface states of TiO_2_. Strategies to improve the stability should be addressed in future studies.

## Author contributions

P. Dash: conceptualisation, methodology, investigation, and writing and editing the original manuscript; Y. Zhong: assistance in methodology; A. Yamamoto: methodology and investigations; D. Takami: analysis and investigations; H. Yoshida: conceptualisation, supervision, methodology, writing and editing the original manuscript, and funding acquisition.

## Conflicts of interest

There are no conflicts to declare.

## Supplementary Material

SC-017-D5SC09539E-s001

## Data Availability

Supplementary information (SI) is provided for this work and available as a pdf file along with the main text. Supplementary information: illustrations are provided for the mechanism and experimental conditions in Fig. S1 and S2. Additional data of XRD, XANES linear component fitting analysis, UV-vis DRS, XANES, EXAFS, TEM, STEM, STEM-EDS, spectral distribution of the Xe lamp, Raman spectra, XRF and XAFS curve fitting analysis are shown in figures from S3, to S9, S11, S12, S16, S17, Table S1, and Table S2 respectively. Additional results of reaction tests are provided in Fig. S10, S13 to S15, and S18, respectively. Further information regarding methods, software and calibrations can be obtained upon request. See DOI: https://doi.org/10.1039/d5sc09539e.
