# Supplementary material for: Nonoxidative coupling of methane to ethane over a Pd–Bi deposited titania photocatalyst in a flow reactor
Source: Chem Sci. 2026 Jan 27;17(12):6208–19. doi: 10.1039/d5sc09539e (PMC12869992; doi:10.1039/d5sc09539e)
Supplement: SC-017-D5SC09539E-s001 [file SC-017-D5SC09539E-s001.pdf]

Supplementary Information (SI) for Chemical Science.
This journal is © The Royal Society of Chemistry 2026

Supporting Information

Nonoxidative coupling of methane to ethane over a Pd–Bi deposited titania photocatalyst in a flow reactor

Preetam Dash, [a] *Yuan Zhong*,[a] *Daichi Takami*,[a] *Akira Yamamoto* [b] *and Hisao Yoshida*[a]*

[a] Graduate School of Human and Environmental Studies, Kyoto University, Yoshida Nihonmatsu-cho, Sakyo-ku, Kyoto 606-8501, Japan.

[b]Department of Applied Chemistry, Faculty of Science and Engineering, Kindai University, 3-4-1 Kowakae, Higashiosaka, Osaka 577-8502, Japan

*Corresponding: yoshida.hisao.2a@kyoto-u.ac.jp

CONTENTS

Figure S1. Expected mechanism of the photoreduction of Pd^{2+} and Bi^{3+} cations over anatase TiO_2.

Figure S2. Experimental setup for the photocatalytic NOCM reaction test using a flow reactor.

Figure S3. The comparison of the XRD pattern of 7Pd-7Bi/TiO_2 with (A) PdO and Bi_2O_3 and (B) intermetallic compounds of Pd and Bi.

Figure S4. The linear component fitting of the Pd K edge XANES spectra of 0.1Pd/TiO_2.

Figure S5. The linear component fitting of the Pd K edge (A) and Bi L_3 (B) XANES spectra of 0.1Pd-0.1Bi/TiO_2.

Figure S6. The Kubelka-Munk spectra of the fresh and used 0.1Pd/TiO_2 sample.

Figure S7. The Bi L_3 XANES spectra.

Figure S8. FT of Bi L_3 EXAFS of 7Pd-7Bi/TiO_2 with references.

Figure S9. (A) TEM images of the 0.1Pd-0.1Bi/TiO_2 sample and (B) Fast Fourier Transform of a detected dark contrast section of the TEM.

Figure S10. (A) Photocatalytic NOCM over bare TiO_2 and (B and C) the images of the TiO2-loaded cell before and after the reaction test.

Figure S11. STEM images of 0.5Pd-0.33Bi/ Al_2O_3.

Figure S12. STEM-EDX analysis of 0.5Pd-0.33Bi/Al_2O_3 sample.

Figure S13. Production rate at 5.5 h on stream for 70%(0.2Pd-0.14Bi/Al_2O_3) + 30%(TiO_2) mounted on the cell by different methods.

Figure S14. Production rate at 5.5 h on stream for two blended catalysts.

Figure S15. Time courses of ethane (black) and hydrogen (red) production rates over the 0.1Pd-0.1Bi/TiO_2 prepared by the DP-H_2 reduction method.

Figure S16. The spectral distribution of the Xe lamp employed.

Figure S17. The Raman spectra for the 0.1Pd-0.1Bi/TiO_2 before and after the 72h stability test.

Figure S18. The 72h stability test for the (A) 0.5Pd/TiO_2 and (B) 0.5Pd-0.5Bi/TiO_2.

Table S1. Loading amount of Pd-Bi in the sample determined by XRF.

Table S2. The curve fitting results of Pd-K edge FT-EXAFS spectra.

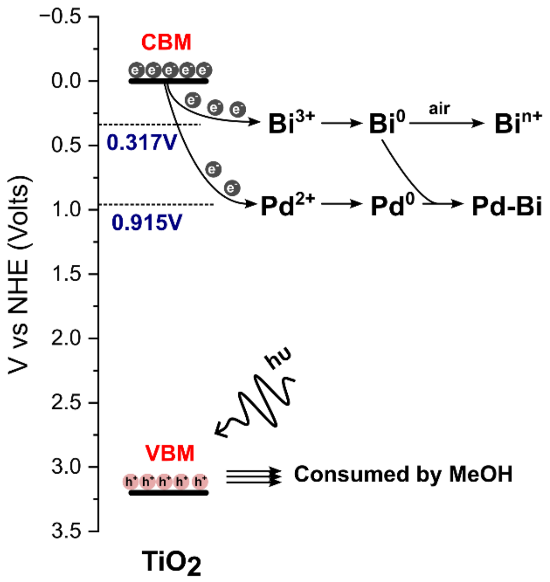

Figure S1. Expected mechanism of the photoreduction of Pd^{2+} [1,2] and Bi^{3+} [3] cations over anatase TiO_2.[4]

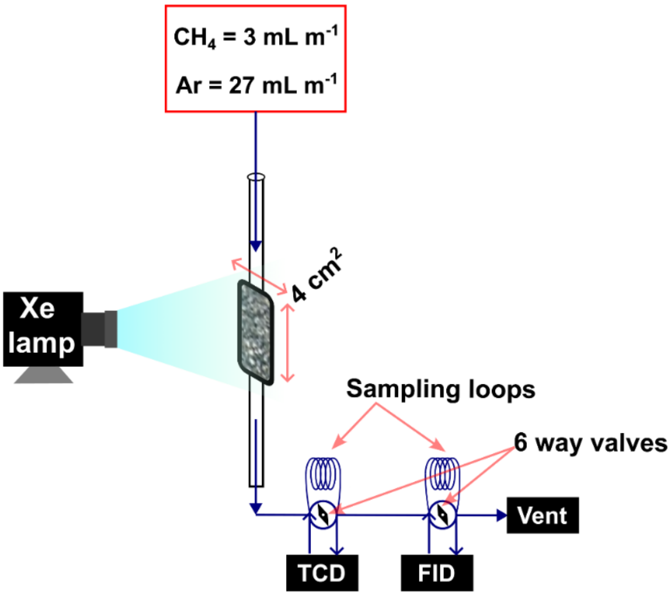

Figure S2. Experimental setup for the photocatalytic NOCM reaction test using a flow reactor.

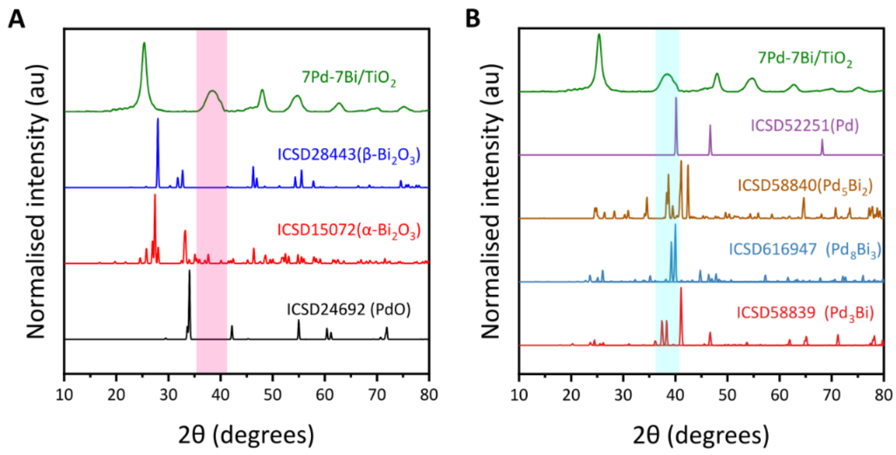

Figure S3. The comparison of the XRD of 7Pd-7Bi/TiO_2 pattern with (A) PdO and Bi_2O_3 and (B) inter-metallic compounds of Pd and Bi. The reference XRD data was obtained from the Inorganic Crystal Structure Database (ICSD).

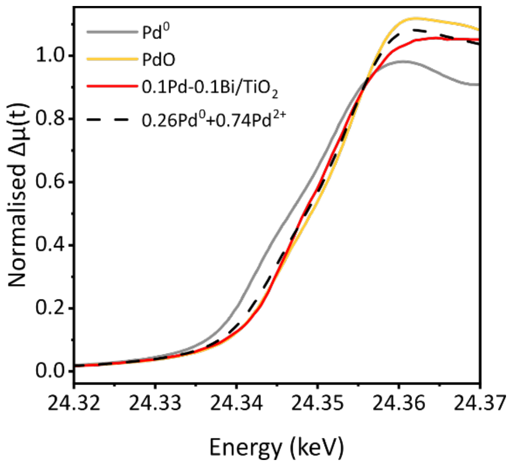

Figure S4. The linear component fitting of the Pd K edge XANES spectra of 0.1Pd/TiO_2.

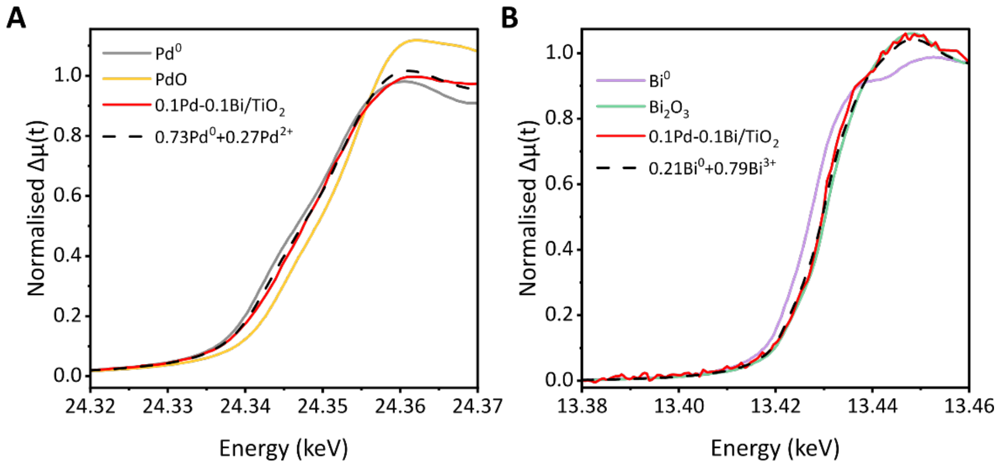

Figure S5. The linear component fitting of the Pd K edge (A) and Bi L_3 (B) XANES spectra of 0.1Pd-0.1Bi/TiO_2.

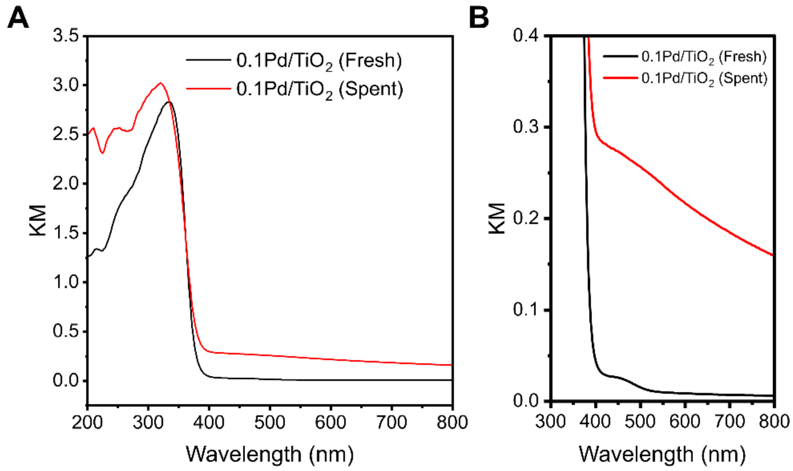

Figure S6. The Kubelka-Munk plot of the UV-Vis DRS spectra of fresh and used 0.1Pd/TiO_2 sample.

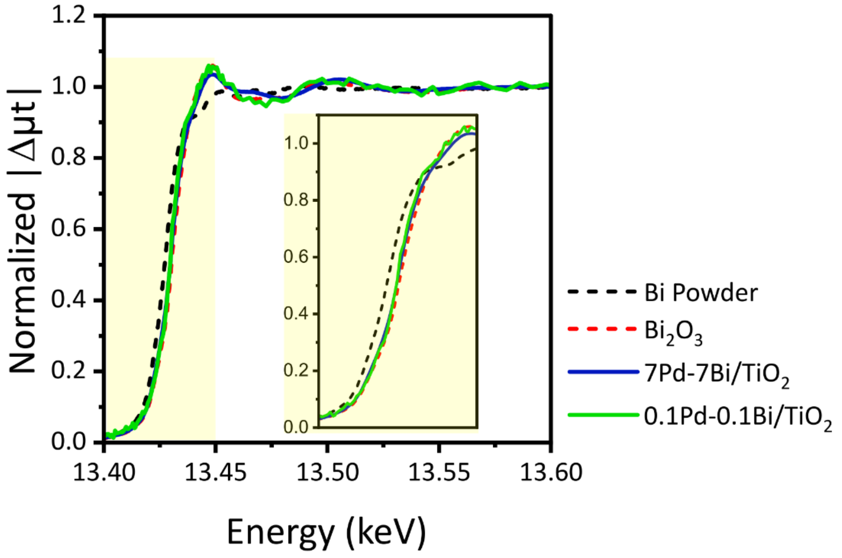

Figure S7. The Bi L_3-edge XANES spectra of samples and references.

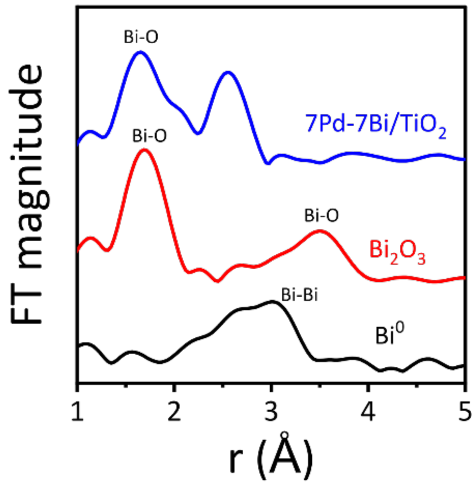

Figure S8. FT of Bi L_3-edge EXAFS for the 7Pd-7Bi/TiO_2 sample and references.

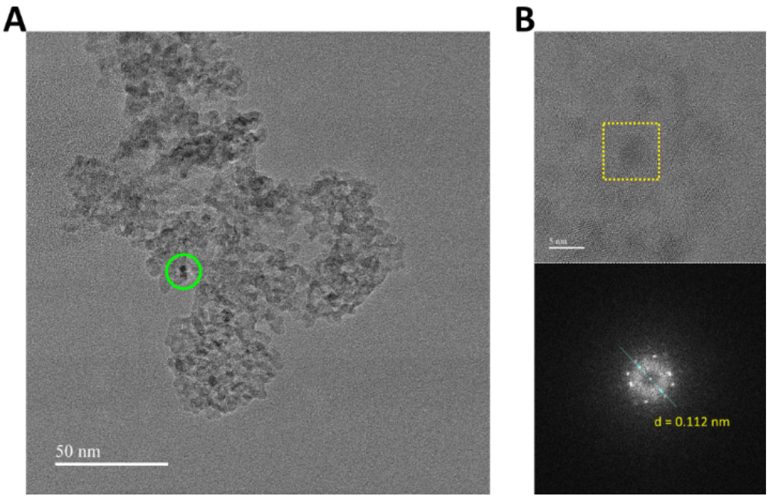

Figure S9. (A) A TEM image of the 0.1Pd-0.1Bi/TiO_2 sample and (B) Fast Fourier Transform of a detected dark contrast section of the TEM: The 1st picture highlights the section of the TEM image used for FFT, and the 2nd picture is the obtained FFT pattern.

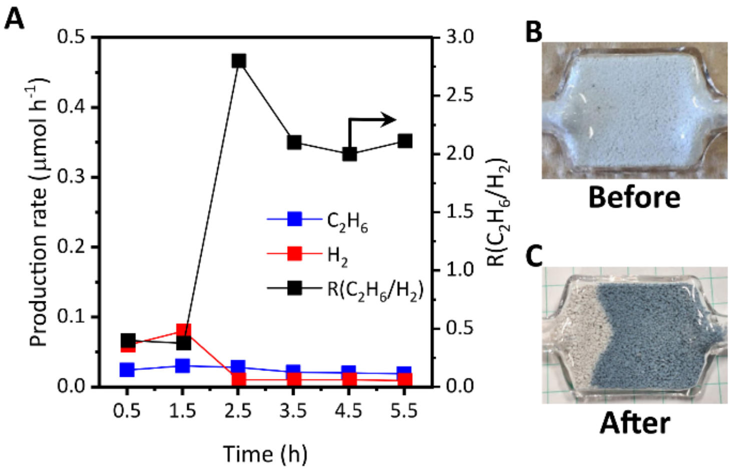

Figure S10. (A) Photocatalytic reaction test for NOCM over the bare TiO_2 (ST01) sample, (B) TiO_2 loaded on the quartz cell before the experiment and (C) after the experiment (the picture was taken after it was exposed to air for 5 minutes after the experiment).

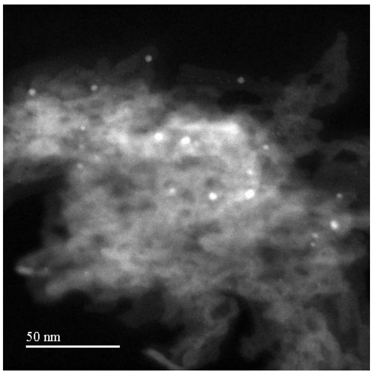

Figure S11. STEM image of 0.5Pd-0.33Bi/Al_2O_3 sample.

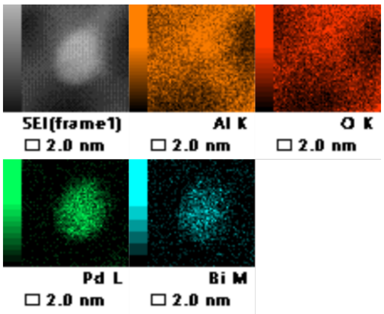

Figure S12. STEM-EDX analysis of 0.5Pd-0.33Bi/Al_2O_3 sample prepared by precipitation method followed by H_2 reduction.

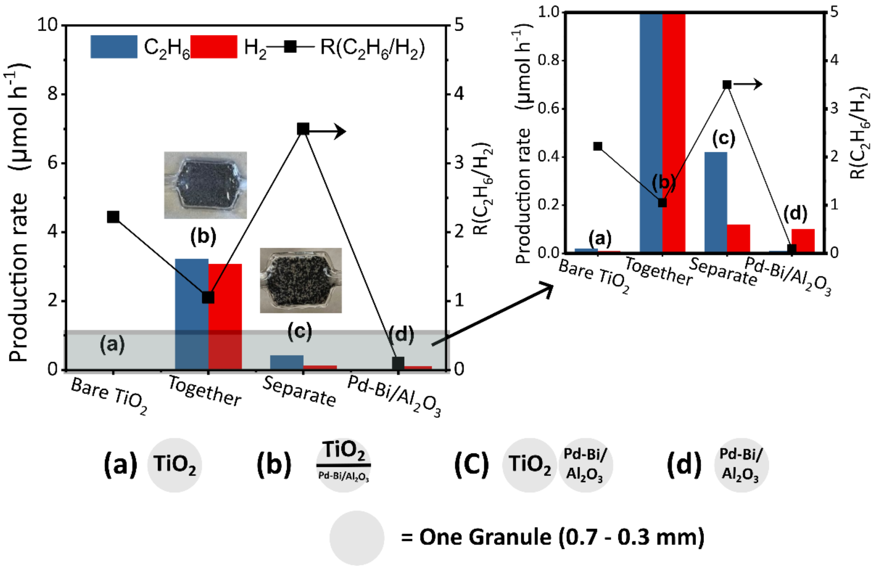

Figure S13. Production rate at 5.5 h on stream for 70%(0.2Pd-0.14Bi/Al_2O_3) + 30%(TiO_2) loaded to the cell by different methods: (a) pure TiO_2, (b) TiO_2 and 0.2Pd-0.1Bi/Al_2O_3

were granulated together, (c) they were granulated separately, and (d) 0.2Pd-0.14Bi/Al_2O_3.

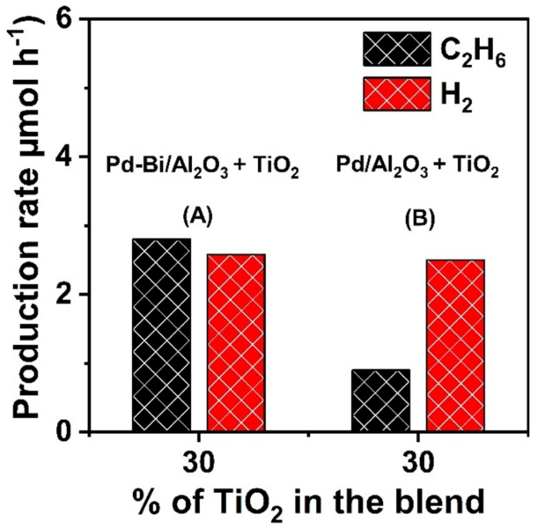

Figure S14. Production rate at 5.5 h on stream for two blended catalysts containing different metal catalysts on alumina support, (A) 70%(0.2Pd-0.14Bi/Al_2O_3) + 30%(TiO_2) and (B) 70%(0.2Pd/Al_2O_3) + 30%(TiO_2).

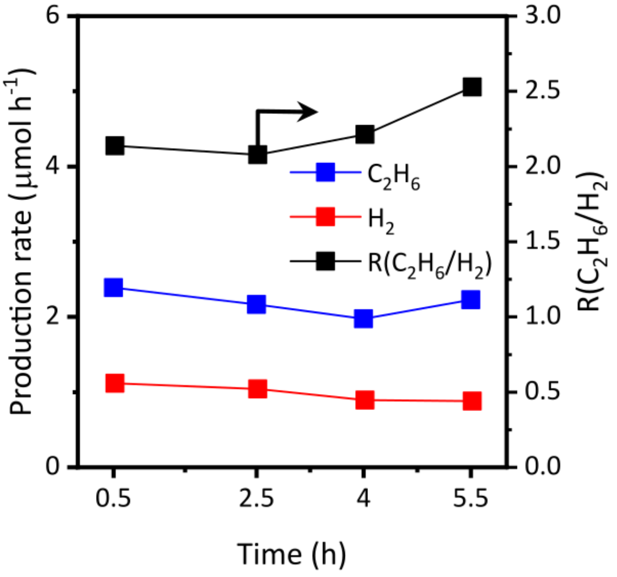

Figure S15. Time courses of ethane (black) and hydrogen (red) production rates over the 0.1Pd-0.1Bi/TiO_2 prepared by the DP-H_2 reduction method.

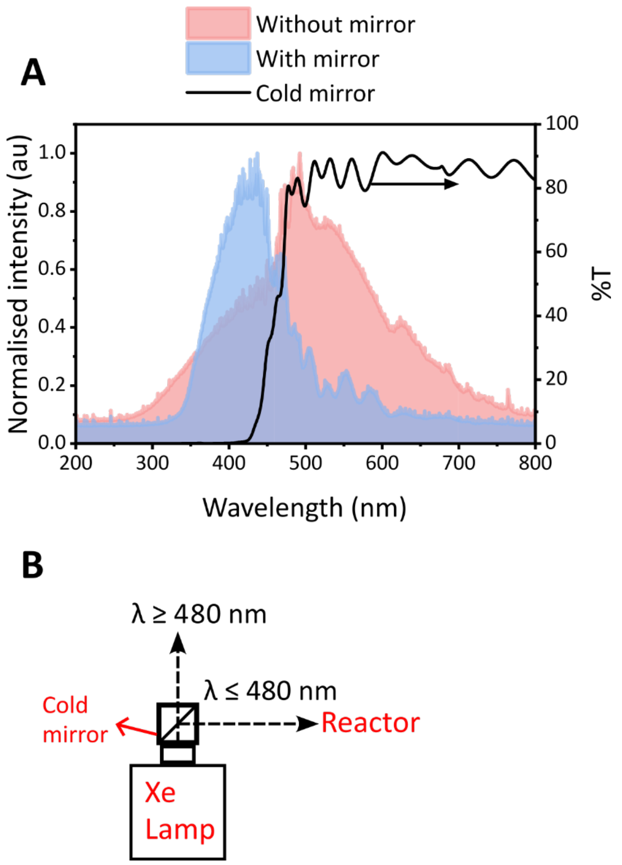

Figure S16. (a) The spectral distribution (left y-axis) of the Xe lamp employed (Red: without cold mirror; Blue: With cold mirror), and the transmittance spectra of the cold mirror employed (right y-axis), (b) A schematic diagram (top view) of the Xe lamp equipped with cold mirror.

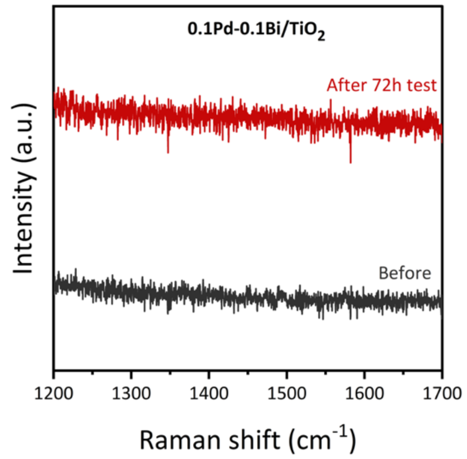

Figure S17. The Raman spectra for the 0.1Pd-0.1Bi/TiO_2 before and after the 72h stability test.

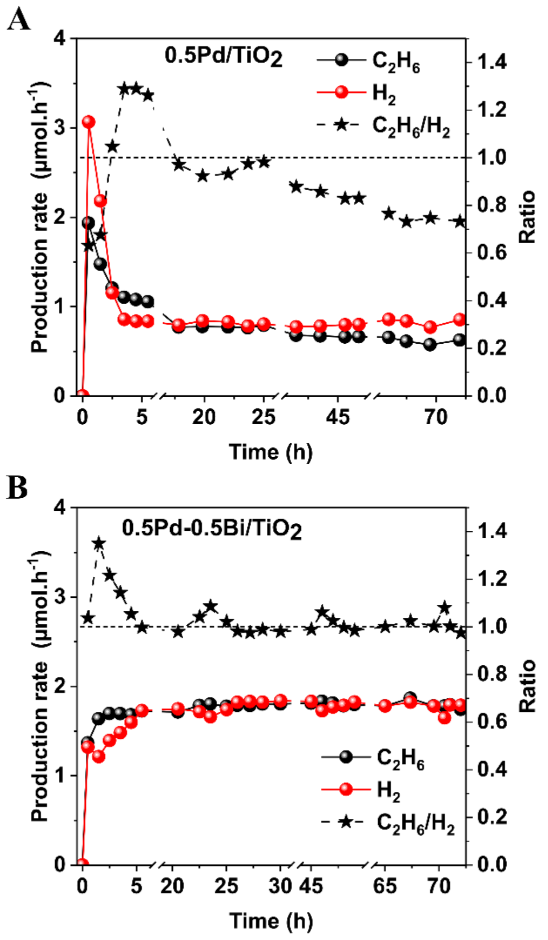

Figure S18. The 72h stability test for the (A) 0.5Pd/TiO_2 and (B) 0.5Pd-0.5Bi/TiO_2.

Table S1. Loading amount of Pd-Bi in the sample determined by XRF

No	Sample	Targeted loading wt%		Actual loading wt%	
		Pd	Bi	Pd	Bi
1	0.1Pd/TiO_2	0.1	0.0	0.103	< 0.0001
2	0.1Pd-0.1Bi/TiO_2	0.1	0.1	0.106	0.110
3	0.1Bi/TiO_2	0.0	0.1	< 0.0001	0.092
4	0.2Bi/TiO_2	0.0	0.2	< 0.0001	0.196
5	0.2Pd/TiO_2	0.2	0.0	0.18	< 0.0001

Table S2. The curve fitting results of Pd-K edge FT-EXAFS spectra.

Sample Name	Shell	CN	R (Å)	dE (eV)	σ^2 (Å^2)	R-factor
Pd foil	Pd-Pd	12	2.74	-7.67	0.0053	0.0094
5Pd/TiO_2	Pd-Pd	5.8 ± 1.3	2.73	-8.34	0.0065	0.0119
7Pd-7Bi/TiO_2	Pd-Pd	5.3 ± 3.2	2.76	-3.02	0.0073	0.0012
	Pd-Bi	4.4 ± 16	2.75		0.026	

References

1. D. H. Templeton, G. W. Watt and C. S. Garner, *J. Am. Chem. Soc.*, 1943, **65**, 1608–1612.

2. N. A. Polotnyanko and I. L. Khodakovskii, *Geochem. Int.*, 2014, **52**, 46–56.

3. S. G. Bratsch, *J. Phys. Chem. Ref. Data*, 1989, **18**, 1–21.

4. A. Fujishima and K. Honda, *Nature*, 1972, **238**, 37–38.
